# Novel Slippery Liquid-Infused Porous Surfaces (SLIPS) Based on Electrospun Polydimethylsiloxane/Polystyrene Fibrous Structures Infused with Natural Blackseed Oil

**DOI:** 10.3390/ijms23073682

**Published:** 2022-03-27

**Authors:** Asma Abdulkareem, Aya E. Abusrafa, Sifani Zavahir, Salma Habib, Patrik Sobolčiak, Marian Lehocky, Hana Pištěková, Petr Humpolíček, Anton Popelka

**Affiliations:** 1 Center for Advanced Materials, Qatar University, Doha P.O. Box 2713, Qatar; asma.alkareem@qu.edu.qa (A.A.); fathima.z@qu.edu.qa (S.Z.); salma.m.habib@hotmail.com (S.H.); patrik@qu.edu.qa (P.S.); 2 Chemical Engineering Program, Texas A&M University at Qatar, Doha P.O. Box 23874, Qatar; aya_abusrafa94@yahoo.com; 3 Centre of Polymer Systems, Tomas Bata University in Zlin, Trida Tomase Bati 5678, 760 01 Zlin, Czech Republic; lehocky@post.cz (M.L.); pistekova@utb.cz (H.P.); humpolicek@utb.cz (P.H.); 4 Faculty of Technology, Tomas Bata University in Zlin, Vavreckova 275, 760 01 Zlin, Czech Republic

**Keywords:** slippery surfaces, electrospinning, plasma treatment, hydrophobic fibrous structures, BSO infusion

## Abstract

Hydrophobic fibrous slippery liquid-infused porous surfaces (SLIPS) were fabricated by electrospinning polydimethylsiloxane (PDMS) and polystyrene (PS) as a carrier polymer on plasma-treated polyethylene (PE) and polyurethane (PU) substrates. Subsequent infusion of blackseed oil (BSO) into the porous structures was applied for the preparation of the SLIPS. SLIPS with infused lubricants can act as a repellency layer and play an important role in the prevention of biofilm formation. The effect of polymer solutions used in the electrospinning process was investigated to obtain well-defined hydrophobic fibrous structures. The surface properties were analyzed through various optical, macroscopic and spectroscopic techniques. A comprehensive investigation of the surface chemistry, surface morphology/topography, and mechanical properties was carried out on selected samples at optimized conditions. The electrospun fibers prepared using a mixture of PDMS/PS in the ratio of 1:1:10 (g/g/mL) using tetrahydrofuran (THF) solvent showed the best results in terms of fiber uniformity. The subsequent infusion of BSO into the fabricated PDMS/PS fiber mats exhibited slippery behavior regarding water droplets. Moreover, prepared SLIPS exhibited antibacterial activity against *Staphylococcus aureus* and *Escherichia coli* bacterium strains.

## 1. Introduction

Biofouling refers to the phenomena of the aggregation and growth of live organisms on wet surfaces and membranes [1,2]. This has a significant impact on liquid transportation, non-fouling marine devices, desalination membranes, and health care devices [3,4,5,6,7,8]. Coating nanoparticles (NPs) on the side in contact with the liquid is one method to prepare an anti-biofouling surface. Ag, TiO_2_, ZnO, and Cu NPs are found to exhibit antimicrobial properties, which primarily kill the microorganisms settled on the surface, acting as a biocide. The synthesis of these NPs is costly and increases the overhead in production for possible commercialization. Furthermore, the performance is reported to decline over time, mainly due to the poor addition of the coat with the substrate. Studies have been conducted on NPs on a graphene oxide (GO) matrix to mitigate some of the concerns encountered with coating pristine metal or metal oxide NPs on membranes. Ag NPs on GO [9,10] and Fe NPs on GO systems [11,12] have been extensively studied in this regard and have been found to demonstrate a synergistic effect. However, they have limited applications, and approaches to fabricate anti-biofouling materials based on different principles are in demand. In health care devices, such as catheters, biofouling control is highly regarded, as this allows pathogens to accumulate and spread diseases. One easy way to overcome this issue is by maintaining the dryness of the surface and sliding away any liquid drops incident on the surface. This approach is more environmentally friendly than biocidal coatings. 

SLIPS [13,14] are the most recent improvement, addressing weaknesses caused by superhydrophobic surfaces [15,16]. Superhydrophobic surfaces have micro/nanostructures on the surfaces that are capable of holding an air layer in between them, preventing the surface from becoming wet by liquid. Conversely, in SLIPS, a lubricating thin film coated on the surface makes sure the liquid droplets on the surface slide away. This concept is inspired by the natural pitcher plant [17], which has a thin lubricating layer on the rough microscopic structures on its peristome. This lubricating film reduces the friction so that any small organism incident on the petal slides to the digestive tract of the plant. 

Polydimethylsiloxane (PDMS) is an elastomeric polymer with low surface energy, high transparency, biocompatibility, and high hydrophobicity [18]. Therefore, PDMS has been reported to exhibit high contact angles >100° and a slippery effect [19]. This water repellency characteristic is important in fouling/contamination prevention [20]. For this reason, PDMS has been diversely used in many applications, including, but not limited to, membrane fabrication [21], biomedical devices [22], and the fabrication of superhydrophobic and/or self-cleaning surfaces [23]. However, pristine PDMS has poor fouling resistance and weak adhesion to substrates [24]. Therefore, PDMS is typically combined with a second component or a mixture to achieve an amphiphilic surface with biofouling activity [25,26,27]. The surface topographies present on the surface decrease the interfacial bonding and thereby inhibit contaminant attachment [28]. PDMS is typically used as either a dense film or fibrous structure. Fibrous PDMS possess high porosity, a large surface area, and remarkable mechanical properties; therefore, it has much wider applications than PDMS films. Fabrication of fibrous networks can be done using the electrospinning technique. Electrospinning has been employed for decades to produce various materials and has proven to be a simple, versatile, and cost-effective technique for the production of thin fibers of nanometer- to micrometer-sized diameters [29]. This method can be applied using a wide range of materials, ranging from natural and synthetic polymers to polymer composites [30,31,32,33]. PDMS has a low molecular weight, and its chain entanglements are typically not enough to provide sufficient crosslinking to form fibrous filaments. For this reason, PDMS is typically incorporated with another carrier polymer to form electrospun fiber mats. Correspondingly, polystyrene (PS) is a suitable candidate for this application, representing an amorphous, transparent polymer with good stiffness and high resistance to electrical forces. PS fiber mats are applied in many industrial aspects, including as ion exchangers [34], in filtration [35] for water purification and some other membrane applications, in medical uses as an enzyme immobilizer [36], in tissue engineering [37,38], in packaging, and as insulation [33]. Typically, two methods are employed to produce electrospun fiber mats: (1) solvent spinning and (2) melt spinning. By far, dissolving the polymer using appropriate solvents is the more widely used method since it is a more adjustable technique. Moreover, the diameter of melt-spun fibers ranges from about 5 µm to 200 µm, whereas electrospun fibers can have diameters in the nanometer range [39]. In the electrospinning process, a polymer solution is placed in a syringe/needle that is subjected to an external electric field applied at the end of the needle [40]. A polymer droplet (known as a Taylor cone) is formed, and when the charge on the surface becomes higher than the surface tension, continuous polymer jets are ejected from the tip of the syringe, which splits into bundles of smaller fibers on a collector surface. The produced thin fibers have a very high surface-to-volume ratio and a high number of fibrous pores [30,32]. Such a technique offers many opportunities to construct controllable surface properties, such as the fiber’s morphology, chemical composition, and functionality. The properties of the electrospun fibers will depend mainly on the polymer’s molecular weight, viscosity, and conductivity [41]. To produce uniform, bed-free fibers, several process parameters need to be adjusted during the electrospinning process, including the distance between the syringe and the collector, applied voltage, and solution concentration. An increase in the voltage beyond a critical value typically results in the formation of beads and an increase in the fiber diameter due to the increase in the jet velocity [42]. Moreover, when the polymer solution concentration is low, the entanglements of the polymeric chains will break into small fragments before reaching the collector [43]. Similarly, the distance between the needle tip and the collector plays a vital role in the properties of the electrospun fibers, specifically the morphology. The surface morphology mainly depends on the deposition time and evaporation rate; hence, a proper distance is required to produce uniform fibrous matrices [44]. The fibers can be tailored depending on the properties/specifications required for their usage.

In this study, novel SLIPS have been fabricated by electrospinning polystyrene PS/PDMS fiber mats on polyethylene PE and polyurethane PU substrates with subsequent BSO infusion. BSO is commonly used in medicine because it demonstrates antimicrobial, anti-inflammatory and antioxidant effects and no cytotoxicity towards human peripheral blood mononuclear cells [45]. The process conditions were optimized to yield a homogenous fibrous mat with the desired properties. The anti-wetting/hydrophobic properties of the prepared fibers, as well as morphology, mechanical properties, and chemical composition, have been thoroughly analyzed. The expected end-use of these SLIPS is on health care products made up of PE or PU. In addition, a biocompatible lubricant like BSO with its natural character and antimicrobial properties, containing 32 compounds, including 9-eicosyne (63.04%), linoleic acid (13.48%) and palmitic acid (9.68%) as major constituents [46,47], in addition to its multiple medicinal properties encourages the implementation of such systems in commercial practice. Antibacterial properties of prepared SLIPS on PU and PE have been investigated and shown to exhibit significant antibacterial activity against Gram-positive *S. aureus* and Gram-negative *E. coli* bacteria. 

## 2. Results and Discussion

### 2.1. Fiber Mat Characterization

#### 2.1.1. Surface Morphology/Topography Analysis

**Figure 1:**
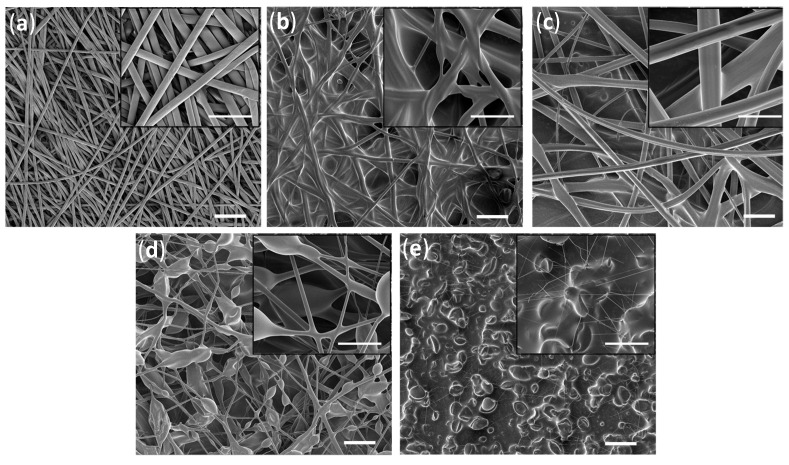
SEM images of: (**a**) neat PS 10%; PS/PDMS fiber mats produced on Al: (**b**) PS/PDMS/THF—1:1:8 (g/g/mL), (**c**) PS/PDMS/THF—1:1:10 (g/g/mL), (**d**) PS/PDMS—0.5:1:10 (g/g/mL), and (**e**) PS/PDMS—1.5:1:10 (g/g/mL). The scale bar is equal to 30 µm.

Electrospun fiber mats composed of PDMS and PS were utilized to create hydrophobic porous structures for oil infusion. The use of PS in THF with a concentration of 10% *w*/*v* (Figure 1a) led to the creation of well-shaped thick fibers. A combination of PDMS with PS overcomes problems with the electrospinning process of pure PDMS [48]. In order to improve the fiber mats’ production processes, several concentrations of the PS/PDMS/THF solution were tested (conditions are provided in Table 1). Using a 1:1:8 ratio of PS/PDMS/THF resulted in the formation of fibers with different diameters and led to the formation of a glue-like fibrous matrix. Nevertheless, SEM images (Figure 1b) of the fibers confirmed the formation of fibrous structures, which proves the good integrity between PS and PDMS in forming a homogenous spinnable polymeric solution. The water contact angle (WCA) showed a hydrophobic behavior (WCA = 124.0 ± 3.6°) (Table 2). Upon increasing the THF solvent content in the polymer mixture (1:1:10 PS/PDMS/THF), the viscosity of the mixture decreased, which resulted in the formation of well-defined fibers. This resulted in fiber mats with larger diameters (rougher surface); hence, the hydrophobicity increased (the contact angle of water for PS/PDMS/THF with a ratio of 1:1:8 was 124 ± 3.6° in comparison with 149.4 ± 3.2° for the PS/PDMS/THF mixture with a ratio of 1:1:10). Decreasing the ratio of PS to PDMS (0.5:1:10) (Figure 1) led to the formation of very thin fibers surrounded by flocs. Moreover, increasing the PS content in the PS/PDMS solution (1.5:1:10) led to the formation of beaded fibers. The formation of flocs might be caused by the low net charge density of the prepared polymer solution [31]. However, the water contact angle was 141 ± 3.8°, which was relatively close to 1:1:10 PS/PDMS/THF (149 ± 3.2°) (Table 2). 

**Figure 2:**
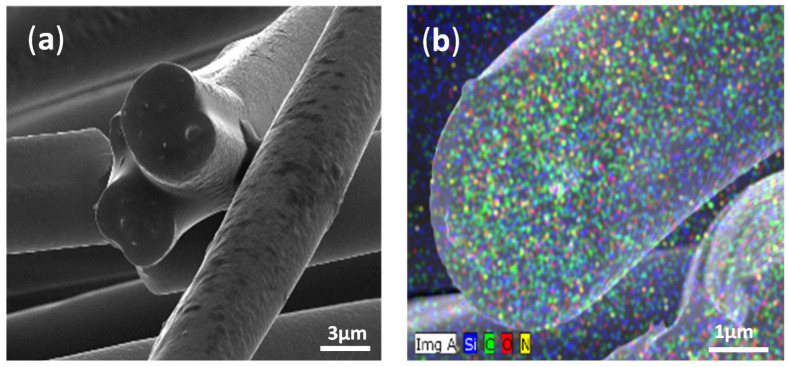
(**a**) SEM image of a cross-section of a fiber. (**b**) SEM-EDX images with mapping of chemical elements of the optimized PS/PDMS 1:1:10 (g/g/mL) fiber mats, including C, O, N, Si.

Figure 2 shows SEM and EDX mapping of the cross-section of electrospun fiber mats containing PS/PDMS/THF—1:1:10 (g/g/mL), confirming the presence of C, O, N and Si elements. Both polymers, PDMS and PS, contribute to C and O elements due to their chemical structures. Distinguishable N and Si elements in Figure 2b confirm the homogenous distribution of PDMS within the electrospun fiber mats. The cross-section view of the fibers in Figure 2a demonstrates that the formed fiber is homogeneous and uniform. The optimized electrospun fibers prepared using the mixture of PS/PDMS in the ratio of 1:1:10 (g/g/mL) using THF solvent on Al foil was used for the formation of fiber mats on the plasma-pre-treated PE and PU substrates, which were used as SLIPS for oil infusion.

**Figure 3:**
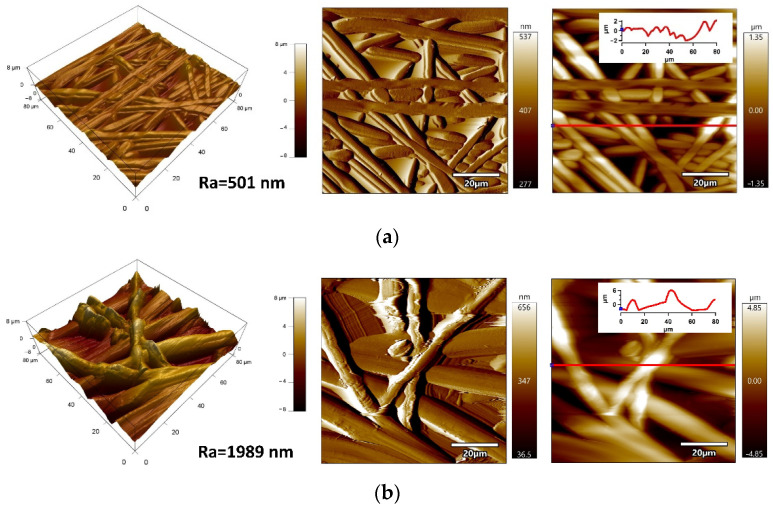
AFM images (from left to right: 3D heighta Amplitude, ZSensor with line profile) of: (**a**) neat PS and (**b**) PS/PDMS 1:1:10 (g/g/mL) on Al substrate.

AFM was performed to provide detailed information about surface morphology/topography of the optimized electrospun fiber mats from the 80 × 80 µm^2^ surface area (Figure 3). This analysis provides information on fiber diameter and the structural features of the fiber mats. It can be observed that the fiber mats produced using neat PS/THF solution (1:10 g/mL) are homogeneous and well-packed; hence, they are expected to possess a relatively small pore size. Smaller pore sizes cause an enhancement in surface hydrophobicity due to the decreased ability of water to penetrate through porous structures. Moreover, it can be remarked that the surface roughness represented by the Ra value (arithmetic mean of line profile) was 501 nm, while surface roughness is a significant factor affecting the hydrophobicity of the surface. The diameter of the fibers can be estimated by measuring the dz of particular peaks in the line profile graphs obtained from ZSensor AFM images. The diameter of PS fibers was approximately 300–700 nm. In the case of the fiber mats fabricated using the polymeric mixture PDMS/PS/THF—1:1:10 (g/g/mL), the fabricated fiber mats excelled with rougher structures, while Ra achieved 1989 nm, caused by the larger diameter of particular fibers around 2–2.5 µm. This increased roughness was responsible for the high water contact angle value (WCA = 149.4 ± 3.2°). The optimized PS/PDMS samples were subsequently used for the preparation of SLIPS on PE and PU substrates, which is discussed later.

#### 2.1.2. Characterization of Mechanical Properties Characterization

The investigation of the mechanical properties was done thoroughly using the nano-indentation measurements by AFM with an MFP-3D Nanoindenter. Hardness and reduced Young’s modulus (E_c_) were measured and calculated as penetration of indenter tip into the fiber mats’ surfaces in different areas and taking an average of 10 points. As the indenter penetration depth was in the range of 2–4 µm, it is assumed that the obtained mechanical properties are attributed to the fiber mats and not only particular fibers. This allowed average values of hardness and E_c_ with standard deviations to be obtained, as shown in Table 3. The relation between stiffness and hardness can vary based on the properties of each material. In practice, the E_c_ of the produced PS fibers prepared in THF solvent was 3.4 ± 0.7 MPa, and their hardness was 0.4 ± 0.2 MPa. In coexistence with PDMS, the E_c_ and hardness of PS/PDMS even increased. The E_c_ value was greater than in the neat PS, achieving a value of 7.2 ± 1.2 MPa, and hardness was increased around 3 times, achieving a value of 1.1 ± 0.3 MPa. Compared to the literature, the mechanical properties of the produced fibers were similar to neat PDMS [49]. 

#### 2.1.3. Chemical Composition Analyses

**Figure 4:**
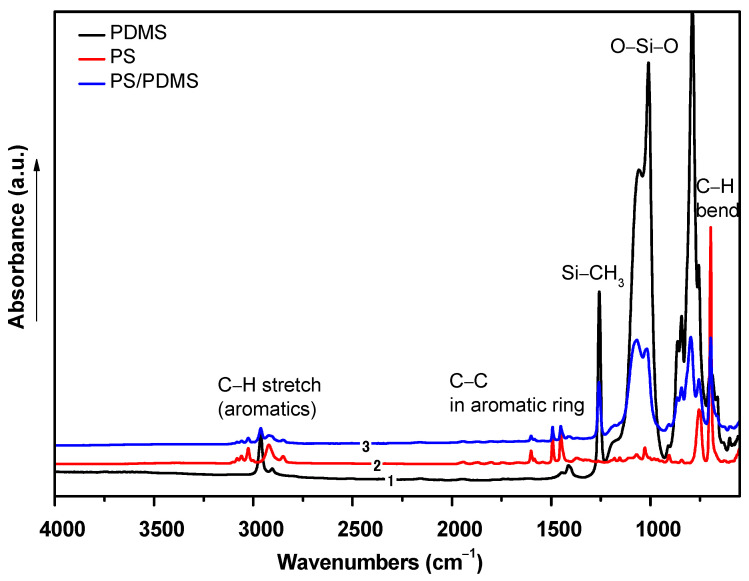
FTIR spectra of PS, PDMS and PDMS/PS electrospun fiber mats.

The chemical composition of the fabricated electrospun fiber mats was analyzed using FTIR-ATR to confirm the presence of certain functional groups. The results are shown in Figure 4. PS in its pure form contains a styrene aromatic ring, which is in agreement with the literature [50]. The main peaks of the pure form shown in Figure 4 are –CH stretching at 3081 cm^–1^–3001 cm^−1^ of the aromatic ring resonation. The stretching of –CH at 2923 cm^−1^ and CH_2_ at 2850 cm^−1^ originates from the asymmetric and symmetric stretching vibrations. The mono-substitution of the benzene ring of styrene occurs at 1943–1728 cm^–1^, and the deformation between –CH_2_ and C–C in the aromatic ring occurs at 1500 cm^–1^. PDMS in its pure form contains three main peaks [51]: an Si–CH_3_ peak at 1270 cm^−1^, O–Si–O at 1160 cm^−1^, and a methyl group –CH at 2980 cm^−1^. When both the polymers were mixed, PS and PDMS shared only the same solution, and their peaks were merged but not reacted. This was observed from the peaks of the PS/PDMS spectrum. The peaks of O–Si–O and –Si–CH_3_ were shown with high intensity at 1160 cm^–1^ and 1270 cm^–1^. Interestingly, the whole peaks of PS were observed in the mixed fibers with lower intensity than the pure form, but no shifting in the wavenumber was observed. The stretching of the peaks of –CH (PS and methyl groups in PDMS) was observed at 3030 cm^–1^ and 2960 cm^–1^. The aromatic C–C was found at 1500 cm^–1^ in the FTIR spectrum of PS/PDMS, and it confirmed the successful incorporation of PS with PDMS.

#### 2.1.4. Adhesion Investigation

**Figure 5:**
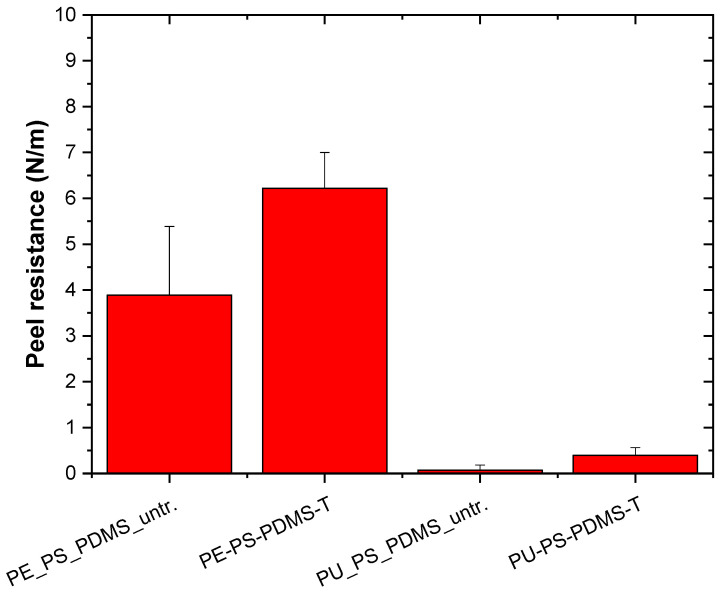
Peel resistance for PS/PDMS electrospun fiber mats of PE and PU.

The plasma treatment was used to improve the wettability and adhesion characteristics of PE and PU substrates prior to the deposition of the PS/PDMS fiber mats. Analyses of adhesion between polymeric substrates and produced electrospun fiber mats were carried out by peel test measurements using Scotch tape. Figure 5 shows peel resistance (peel force per entire width) between PS/PDMS fiber mats and the PE and PU substrates. Peel resistance of untreated PE was relatively low, achieving a value of 3.9 ± 1.5 N/m. The plasma treatment was responsible for an improvement in the adhesion of PE, while peel resistance increased to 6.2 ± 0.8 N/m. More visible differences were observed for PU. The peel resistance of untreated PU was much lower compared with untreated PE, achieving 0.1 ± 0.1 N/m because of the lower surface free energy of PU. Plasma treatment led to a remarkable increase in adhesion, while peel resistance increased to 0.4 ± 0.2 N/m. The improvement of the adhesion of the plasma-treated polymeric surface was caused by the improvement in wettability as a result of changes in the functionalization and roughness [52,53]. 

### 2.2. Characterization of SLIPs

#### 2.2.1. Surface Morphology/Topography Analysis

**Figure 6:**
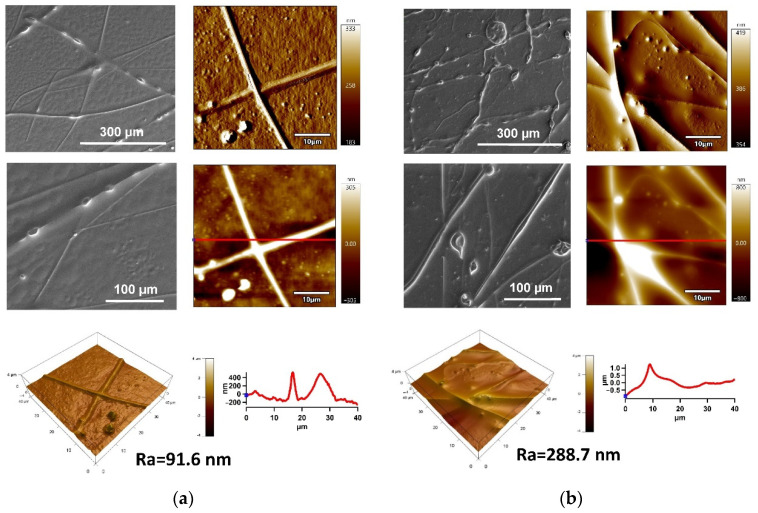
AFM images (from left to right: 3D height, amplitude, ZSensor with line profile) of PS/PDMS/BSO (PS/PDMS/THF—1:1:10 (g/g/mL)) on: (**a**) PE and (**b**) PU substrates.

SEM and AFM techniques were used to analyze fabricated SLIPS on the PE and PU substrates. The surface morphology/topography images of the PE and PU substrates with applied PDMS/PA/BSO are shown in Figure 6. A relatively homogeneous infusion of BSO into the porous structures of PS/PDMS fiber mats was observed by SEM images, while only a few uncovered fibers were present. Further detailed surface morphology/topography characterization of prepared SLIPs was analyzed by AFM from the 40 × 40 µm^2^ surface area. The PS/PDMS/BSO SLIPS fabricated on the PE substrate were characterized by a smoother surface (Ra = 91.6 nm) in comparison with the same fabricated on the PU substrate, while Ra achieved 288.7 nm. Moreover, the line profile revealed the irregularities of SLIPS on the PE substrate, with a maximum of about 785 nm in comparison to the 2085 nm for PS/PDMS/BSO SLIPS fabricated on the PU substrate in the entire surface area.

#### 2.2.2. Slippery Behavior Investigation

**Figure 7:**
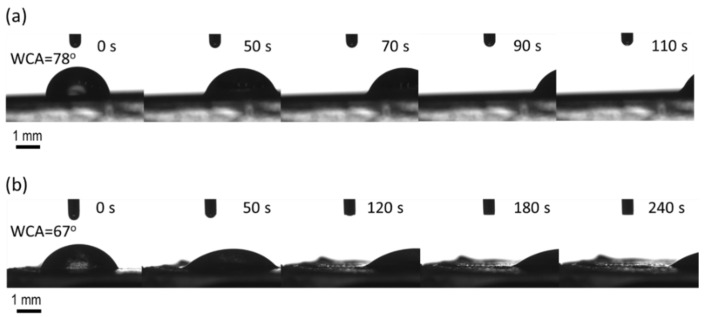
Representative sliding behavior of water droplet at 10° tilting on (**a**) PDMS/PS/BSO-PU and (**b**) PDMS/PS/BSO-PE.

As mentioned, slippery behavior observed in SLIPS provides an environmentally way to combat the adhesion of pathogens or any particle of that sort on a surface. In this concern, PDMS/PS/THF—1:1:10 (g/g/mL) fiber mats fabricated on plasma-treated PE or PU substrates were converted into a slippery surface by modification with natural-based BSO via spin coating. Visual examination of the BSO layer revealed it to be stable and sufficiently wet within the fiber mat substrate. Pathogens are more easily transported by water than any other liquid encountered during daily activities. Hence, water was employed as the model-impinging liquid to evaluate the potential liquid-repellent performance of BSO-infused PDMS/PS fiber mats. PDMS/PS fiber mats on substrate exhibited a WCA of ~149°, which was reduced to ~78° for PDMS/PS/BSO-PU and ~67° with PDMS/PS/BSO-PE. The key requirements of the lubricating liquid (BSO) are to have a higher affinity for the fiber mat surface compared to that of the impinging liquid (water) and for the two liquids to be immiscible [14]. Immiscibility is confirmed by the satisfactory water contact angle values depicted in Figure 7. As can be seen, BSO-infused fiber mats prepared on the PU substrate demonstrated faster sliding behavior than on the PE substrate. Additionally, water droplets of 3 µL, used to avoid the line pinning effect, had a more globular structure on the PU substrate (Figure 7a), and the shape was maintained throughout the sliding period. Conversely, the droplet was more spread on the PE substrate (Figure 7b), as observed by the CCD camera connected to the contact angle measuring device. Precise analysis shows that the translation of the water droplet was similar on both substrates after the initial 50 s. It is also noteworthy that the water droplet was dispensed on the surface at 0°, followed by tilting to 10° at a gentle base to avoid vibrational impacts on the droplet during the sliding stage. At 50 s, the stage reaches a 10° inclination, and the translation of the impinging droplet afterward is distinctly two-fold faster with the BSO-infused fiber mat on the PU substrate. However, PDMS/PS/BSO on both the PU and PE substrates demonstrate favorable sliding behavior with water as the impinging liquid. Thus, the use of a biocompatible lubricant like BSO with multiple medicinal properties encourages the implementation of such systems in commercial practice. 

#### 2.2.3. Antibacterial Activity Investigation

**Figure 8:**
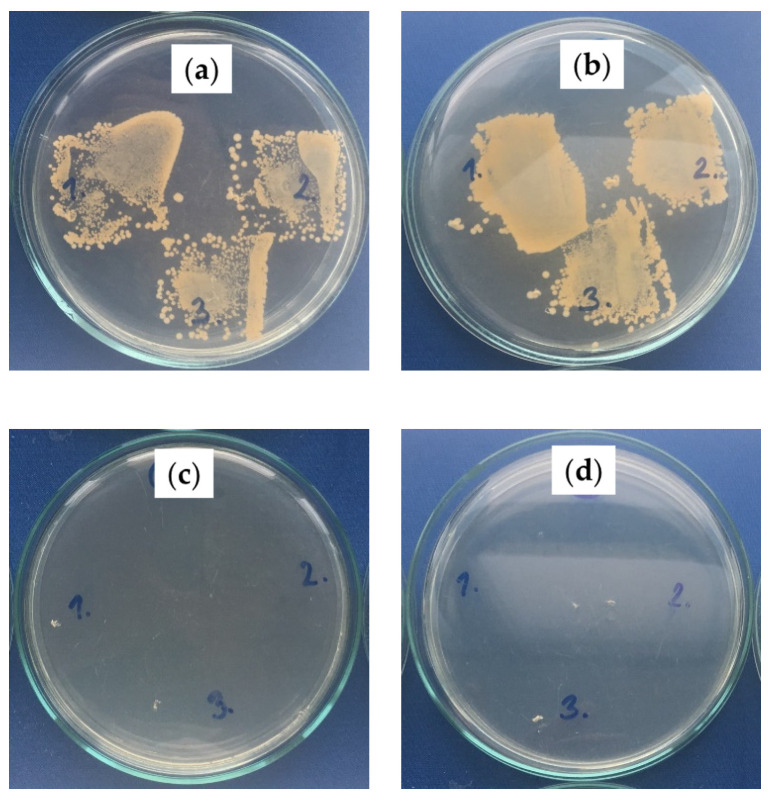
Total bacterial counts on plate-count agar with inoculated *S. aureus*: (**a**) PS/PDMS-PE, (**b**) PS/PDMS-PU, (**c**) PS/PDMS/BSO-PE, (**d**) PS/PDMS/BSO-PU; numbers represent various imprint locations.

**Figure 9:**
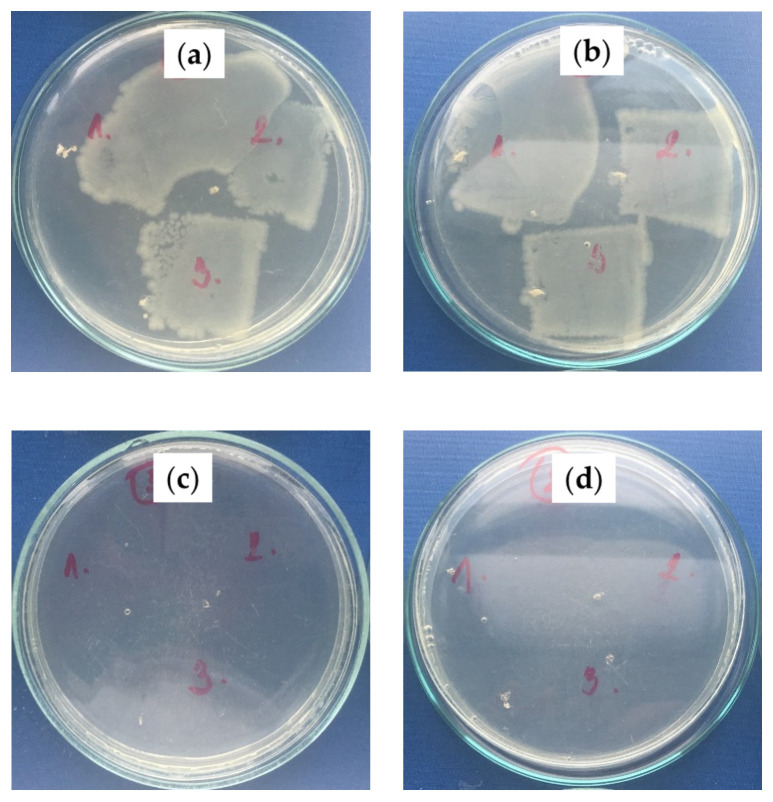
Total bacterial counts on plate-count agar with inoculated *E. coli*: (**a**) PS/PDMS-PE, (**b**) PS/PDMS-PU, (**c**) PS/PDMS/BSO-PE, (**d**) PS/PDMS/BSO-PU; numbers represent various imprint locations.

The antibacterial properties of fabricated SLIPS on the polymeric substrates were evaluated using *S. aureus* (Gram-positive) and *E. coli* (Gram-negative) bacterium strains. Increases in the bacterial colonies of the PS/PDMS samples are summarized in Table 4, and representative photos are shown in Figure 8 and Figure 9 for *S. aureus* and *E. coli*, respectively. The fabrication of PS/PDMS fiber mats on the PE or PU substrate demonstrated poor antibacterial activity against *S. aureus* and *E. coli,* with poor bacterial proliferation inhibition caused by their chemical nature. However, subsequent fabrication of SLIPS using the BSO infusion into the PS/PDMS porous structures led to a significant improvement in antibacterial activity, as BSO has a strong antibacterial effect. PS/PDMS/BSO-PE samples demonstrated an increase of zero bacterial colonies for both *S. aureus* and *E. coli,* and PS/PDMS/BSO-PU samples were characterized by zero colonies for *S. aureus* and zero or single colonies detectable for *E. coli*. This analysis proved the significant antibacterial effect of prepared SLIPS.

## 3. Materials and Methods

### 3.1. Material

For the preparation of the polymeric substrate for modification, commercial-grade low-density polyethylene (LDPE) FE8000 pellets kindly provided by Qatar Petrochemical Company (QAPCO, Doha, Qatar) were used. Compression molding was used to create thin homogeneous films with a thickness of around 0.41 mm using an industrial mounting press machine (Carver, Wabash, IN, USA). To produce a flat surface, the pellets were melted at 160 °C and compressed for 2 min with a force of 2 tons while keeping the temperature constant. Water was used to cool the LDPE films to room temperature. 

Commercial films of polyether-based polyurethane (PU), approximately 0.21 mm thick, were provided by American Polyfilm (American Polyfilm Inc, Branford, CT, USA).

The above-mentioned LDPE and PU films were cleaned with acetone and ethanol, respectively, to remove any dust or potential contamination from the manufacturing process that might impact the surface characteristics, and then air-dried for 20 min at room temperature (RT). 

A polydimethylsiloxane (PDMS) commercial SYLGARD 184-elastomer kit was purchased from Dow Corning (USA), described as two-part, clear, 10:1, RT and heat cure, good strength, UL and Mil Spec.

PS was purchased from Sigma-Aldrich of Mw (350,000 g/mol-350 k), CAS Number 9003-53-6, MDL number MFCD00084450. 

Tetrahydrofuran (THF) and N, N-dimethylformamide (DMF) were purchased from Sigma-Aldrich. Isopropyl alcohol (IP) was purchased from BDH and was anhydrous (≥99% purity) and inhibitor-free. Ultrapure water (Purification System Direct Q3, Molsheim, France) was used to investigate the wettability and slippery properties. Blackseed oil (BSO) (AL Mashreq Int., 125 mL, Doha, Qatar) was utilized as a lubricant.

### 3.2. Procedure

#### 3.2.1. Plasma Treatment

A low-temperature plasma treatment of the PE and PU substrates was used before the preparation of electrospun fibers with infused oils on them. For this purpose, the radio-frequency (RF) plasma system Venus75-HF (Plasma Etch Inc., Carson, CA, USA) working at reduced pressure (~25 Pa) was employed. The reactive species, such as electrons and ions responsible for the treatment of polymeric substrates, were generated by an application of a high voltage at a typical frequency of 13.56 MHz. The nominal power was adjusted to 80 W for both, PE and PU substrates. The applied treatment time based on the previous optimizations was 120 s for PE [52] and 180 s for PU [53].

#### 3.2.2. Electrospinning Procedure

Neat PS fiber mats were fabricated using PS/THF solution with a ratio of 1:10 (g/mL). The PS/PDMS fiber mats were fabricated using a NaBond (Shenzhen, China) electrospinning device. Aluminum foil was placed on a plate or drum and was used as a collector of fiber mats to ensure a conductive substrate. Then, the plasma-treated PE and PU substrates (with improved adhesion as a result of increased wettability) were adhered to the aluminum foil in order to create porous structures on polymeric substrates. For the electrospinning process, 5 mL syringes were used, and the electrospinning time was approximately 2 h in multi-jet mode. The procedure for the fabrication of PS/PDMS fiber mats was carried out at a voltage of 12.5–15 kV, a needle/collector distance of 15 cm, and a flow rate of 2.5 mL/h. PDMS with the curing agent was used in a fixed ratio (10:1), and as a mixture, they were used in different concentrations with THF, as shown in Table 1.

#### 3.2.3. Oil Infusion 

To create a slippery surface, natural-based BSO was infused into the electrospun fiber mats using the spin coating technique. This technique allows the production of a uniform thin film layer by depositing a small drop of BSO onto the center of a substrate and then spinning the substrate at high speed. Moreover, this ensures the removal of oil excess from the substrate. The oil infusion into the electrospun fibers was carried out using Spin coating WS 650-23 (Spincoater-Laurell, North Wales, PA, USA) at a speed of 500 rpm for 60 s.

#### 3.2.4. Surface Energy and Slippery Effect Investigation

The changes in the hydrophobicity and slippery behavior of the prepared electrospun fibers with infused oil treated by plasma or heated were evaluated by static water contact angle measurement. The optical contact angle measuring system OCA35 (DataPhysics, Filderstadt, Germany) was used for this study, which is equipped with a high-resolution CCD camera. Ultrapure water was used as a testing liquid. A water droplet of 5 μL was dispensed at ambient air conditions. The contact angle was calculated approximately after 3 s (when a thermodynamic equilibrium was reached between the liquid and the sample interfaces), and then the slippery effect of the created electrospun fiber mats with infused BSO was analyzed using a tilted stage at an angle of 10°. The water droplet was placed at 0° on the sample, and 10° was reached by slowly tilting the whole equipment with the stage and camera (~50 s time duration). A minimum of 5 readings were recorded from different surface areas in order to obtain average values of contact angles and standard deviations. Moreover, a homogeneity of fabricated SLIPS was first confirmed by analyzing different surface areas in terms of sliding behavior, and then representative images were recorded.

#### 3.2.5. Surface Morphology/Topography Analysis

The surface morphology after plasma treatment was studied using scanning electron microscopy (SEM). SEM microscope (Nova NanoSEM 450, FEI, Hillsboro, OR, USA) was used to obtain 2D images of the analyzed surfaces. To get higher resolution images, thin Au layers a few nanometers thick were sputter-coated onto prepared fiber mats.

Detailed information about 3D changes in the surface topography of the fibers was obtained using atomic force microscopy (AFM). The AFM device MFP-3D (Asylum Research, Oxford Instruments, Santa Barbara, CA, USA) was employed in these experiments. Scanning was carried out under ambient conditions by a silicon probe (Al reflex-coated Veeco model—OLTESPA, Olympus) in the tapping mode in air (AC mode), allowing images to be obtained from a surface area of 20 × 20 µm^2^. A homogeneity of different surface areas was first pre-checked by scanning different surface areas, and subsequently, representative high-resolution images were recorded. Moreover, the surface roughness parameter (Ra) representing the arithmetical mean height was evaluated.

#### 3.2.6. Investigation of Mechanical Properties 

To identify changes in the mechanical properties of the prepared fiber mats, a nanoindentation technique was used. This technique allows the measuring of indentation depths on the surface of thin sections by an application of very low forces to the indentation tip. The MFP-3D Nano Indenter (Asylum Research, Oxford Instruments, Santa Barbara, CA, USA) using a standard diamond three-side pyramidal-shaped Berkovich indenter tip (TB15773) with a tip radius ~100 nm and a spring constant of 3940 N/m (calibration with sapphire standard) was applied. Different areas (10 indentations in order to evaluate average values and standard deviations of mechanical properties) of fiber mats were examined with a loading force from 0 to 50 μN at a loading rate of 10 μN/s. The indentation force was kept for 5 s, and then unloading from 50 μN to 0 μN occurred at an unloading rate of 10 μN/s. The hardness (H) (Equation (1)) and the reduced modulus (E_c_) (Equation (2)) of prepared fibers were then calculated using the Oliver–Pharr method [54], considering an elastic behavior during the initial stages of unloading.

(1) $$H=\frac{P_{\max}}{A}$$

where P_max_ is the peak indentation load and A is the projected area of the hardness impression.

(2) $$\frac{1}{E_{c}}=\frac{\left(1-\nu^{2}\right)}{E}+\frac{\left(1-\nu_{i}{}^{2}\right)}{E_{i}}$$

where E and ν are Young’s modulus and Poisson’s ratio for the specimen, and E_i_, and ν_i_ are the same parameters for the indenter tip.

#### 3.2.7. Adhesion Investigation

A standard test technique ASTM D6862 was used to investigate the adhesion properties at the macro level. A Peel Tester LF-Plus (Lloyd Instruments, West Sussex, UK) equipped with a 1 KN cell was used to test the peel resistance at 90° of peeling between the PS/PDMS fiber mats and the PE or PU substrate. To achieve a constant peeling, the adhesive joints (19 mm width) were delaminated at a rate of 10 mm/min. To achieve average values for peel resistance, six independent measurements were taken.

#### 3.2.8. Chemical Composition Analyses

To assess the chemical composition changes in plasma-treated fiber mats and their surfaces, Fourier-transformed infrared spectroscopy with an attenuated accessory (FTIR-ATR) was used. The Spectrum 400 (Perkin Elmer, Waltham, MA, USA) was used to identify the functional groups introduced after the plasma treatment and to characterize the chemical composition of prepared fiber mats. All measurements were obtained through eight scans with a resolution of four after background subtraction. Qualitative information for the absorbance of chemical groups in the middle infrared region (4400–550 cm^−1^) was obtained.

#### 3.2.9. Antibacterial Tests

The antibacterial activity of modified polymeric materials was evaluated using ISO 22196, which was adapted to investigate the antibacterial effects of the produced samples. The samples were inoculated with standardized bacterial suspensions of *Staphylococcus aureus* (CCM 4516—2.3 × 10^5^ cfu/mL) *or Escherichia coli* (CCM 4517—7.1 × 10^6^ cfu/mL) 100 mL and covered with ethanol-disinfected polypropylene foil with dimensions of 20 mm by 20 mm. Following that, the infected samples were incubated for 24 h at 100% relative humidity and 35 °C. After removing the polypropylene foil, samples were imprinted on agar (three times from various regions) with lecithin 0.7 g/L and Tween 80 5 g/L as neutralizers and incubated at 35 °C for 24 h. For each sample, antibacterial activity was analyzedthree times in order to obtain statistical information. The number of bacterial colonies was then assessed on a scale of 0–5, with 0 being the best antibacterial efficacy without bacteria growing and five representing no antibacterial effect with bacteria growing.

## 4. Conclusions

SLIPS have been fabricated on polyethylene PE and polyurethane PU substrates by electrospinning polystyrene PS/PDMS fiber mats with subsequent BSO infusion. Various surface morphologies, including beads with different sizes and shapes, bead-on-string structures, as well as well-defined fibers with various diameters, were formed during the electrospinning process. Results showed that all the prepared fibrous structures showed high resistance to wetting due to their surface hydrophobicity (contact angle > 100°). Upon using PS in PDMS as a carrier polymer, well-defined bead-free fibers were obtained by a direct electrospinning solution containing PS/PDMS/THF (1:1:10). Plasma treatment acted as an adhesion promoter between the polymeric substrate and PS/PDMS electrospun fiber mats, which was confirmed by peel resistance measurements. The subsequent infusion of natural BSO into the porous structures caused slippery behavior, as was confirmed by sliding angle measurements at 10° of titling angle. Moreover, the antibacterial properties of prepared SLIPS on PU and PE have been investigated and found to exhibit significant antibacterial activity against Gram-positive *S. aureus* and Gram-negative *E. coli* bacteria.

## Figures and Tables

**Table 1:** Parameters/conditions for preparation/electrospinning of PS/PDMS.

Polymer Mixture		Solvent
PS	PDMS	THF
(g)	(g)	(mL)
0.5	1	10
1	1	10
1.5	1	10
1	1	8
1	1	6

**Table 2:** Properties of prepared PS/PDMS fiber mats.

Polymer	WCA (°)	WCA Image
0.5 g:1 g PS: PDMS in 10 mL THF	127.4 (±2.4)	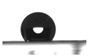
1 g:1 g PS: PDMS in 10 mL THF	149.4 (±3.2)	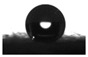
1 g:1 g PS: PDMS in 8 mL THF	124.0 (±3.6)	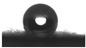
1.5 g:1 g PS: PDMS in 10 mL THF	141.3 (±3.8)	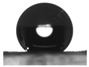

**Table 3:** The reduced Young’s modulus E_c_ and the hardness of the electrospun fibers.

Sample	E_c_ (MPa)	SD	Hardness (MPa)	SD
PS	3.4	0.7	0.4	0.2
PS/PDMS	7.2	1.2	1.1	0.3

**Table 4:** Antibacterial activity of samples.

Sample	Increase in Bacterial Colonies *	
	*S. aureus* CCM 4516	*E. coli* CCM 4517
PS/PDMS-PE (no oil)	4–5, 4, 4	5, 5, 5
PS/PDMS-PU (no oil)	4–5, 4–5, 4–5	5, 4–5, 5
PS/PDMS/BSO-PE	0, 0, 0	0, 0, 0
PS/PDMS/BSO-PU	0, 0, 0	0, 0–1, 0–1

* The scale for assessing the growth of bacterial colonies: 0—without growth, 1—detectable amount (single colony), 2—detectable amount (combined colony), 3—second imprint—distinguishable colonies, third imprint can be detected, 4—third imprint—distinguishable colonies, 5—overgrown—continuous growth.

## Data Availability

This research follows MDPI Research Data Policies.
